# WHEN AND HOW PERCEIVED CONTROL BUFFERS AGAINST COGNITIVE DECLINES: A MODERATED MEDIATION ANALYSIS

**DOI:** 10.1093/geroni/igae098.0507

**Published:** 2024-12-31

**Authors:** Jeremy Hamm, Margie Lachman, Katherine Duggan, Jacqueline Mogle, Ryan McGrath, Kelly Parker, Laura Klepacz

**Affiliations:** North Dakota State University, Fargo, North Dakota, United States; Brandeis University, Waltham, Massachusetts, United States; North Dakota State University, Fargo, North Dakota, United States; Clemson University, Clemson, South Carolina, United States; North Dakota State University, Fargo, North Dakota, United States; North Dakota State University, Fargo, North Dakota, United States; North Dakota State University, Fargo, North Dakota, United States

## Abstract

People vary widely in their rates of age-related cognitive decline. Slower rates of decline have been linked to individual differences in perceived control, but less is known about how (mechanisms) and at what stage in the lifespan (moderators) this modifiable psychological resource is linked to preserved cognitive functioning. Our study examined changes in light physical activity (LPA) as an unexplored mechanism that may link changes in two facets of perceived control (personal mastery, perceived constraints) to 9-year trajectories of cognitive aging. We also examined whether mediated pathways were moderated by age (i.e., differed across the adult lifespan). We analyzed 9-year data from the Midlife in the United States Study (n=2,456; age= 56±11 years) using autoregressive mediation and moderated-mediation models. Mediation models showed that changes in personal mastery and perceived constraints predicted changes in LPA (βs=.06-.08, ps<.01), which in turn predicted 9-year changes in episodic memory and executive functioning (βs=.05-.13, ps<.02). Moderated-mediation models showed that, for episodic memory, the mediated pathways were strongest in old age and emerged only for constraints: For only older adults, declines in constraints predicted less decline in LPA (β=.11, p<.001), which in turn predicted slower declines in episodic memory (β=.05, p=.001). Results were consistent in sensitivity analyses that controlled for levels and changes in moderate-to-vigorous physical activity. Our findings inform lifespan theories of control and provide initial evidence that changes in a largely overlooked health behavior (LPA) may underlie the influence of perceived constraints on cognitive functioning, with this pathway becoming more pronounced in late life.

